# Balbiani body of basal insects is potentially involved in multiplication and selective elimination of mitochondria

**DOI:** 10.1038/s41598-024-58997-6

**Published:** 2024-04-09

**Authors:** Malgorzata Sekula, Waclaw Tworzydlo, Szczepan M. Bilinski

**Affiliations:** Department of Developmental Biology and Invertebrate Morphology, Institute of Zoology and Biomedical Research, Faculty of Biology, Jagiellonian University in Krakow, Gronostajowa 9, 30-387 Kraków, Poland

**Keywords:** Oogenesis, Mitochondria, Entomology

## Abstract

Oocytes of both vertebrates and invertebrates often contain an intricate organelle assemblage, termed the Balbiani body (Bb). It has previously been suggested that this assemblage is involved in the delivery of organelles and macromolecules to the germ plasm, formation of oocyte reserve materials, and transfer of mitochondria to the next generation. To gain further insight into the function of the Bb, we performed a series of analyses and experiments, including computer-aided 3-dimensional reconstructions, detection of DNA (mtDNA) synthesis as well as immunolocalization studies. We showed that in orthopteran *Meconema meridionale*, the Bb comprises a network of mitochondria and perinuclear nuage aggregations. As oogenesis progresses, the network expands filling almost entire ooplasm, then partitions into several smaller entities, termed micro-networks, and ultimately into individual mitochondria. As in somatic cells, this process involves microfilaments and elements of endoplasmic reticulum. We showed also that at least some of the individual mitochondria are surrounded by phagophores and eliminated via mitophagy. These findings support the idea that the Bb is implicated in the multiplication and selective elimination of (defective) mitochondria and therefore may participate in the transfer of undamaged (healthy) mitochondria to the next generation.

## Introduction

The Balbiani body (Bb) has been described in the oocytes of nearly all examined animal species^1,2^. As a rule, the Bb is a transient complex of diverse organelles located next to the oocyte nucleus (germinal vesicle) and often attached to its envelope. Morphology of the Bb may differ even in related species^3^ and appears dynamic throughout its morphogenesis, i.e., its rise in early oocytes, subsequent growth, and ultimate fragmentation/degradation^1–6^. Despite this variability, the Bbs always contain two constant elements: mitochondria and accumulations of electron-dense fibrillo-granular material, the nuage (the function and composition of the nuage are discussed in^2,7,8^). In addition to these indispensable elements, the Bbs may also comprise centrioles, cisternae and/or vesicles of endoplasmic reticulum (ER), Golgi complexes (GCs), lipid droplets (LDs), and cytoskeletal fibers^1,2,9^. The formation of the Bb, i.e., the integration of various cellular components into a single intricate complex is not fully understood. Recent analyses indicate that the Bb (as well as other organelles not surrounded by a limiting membrane) represents a biomolecular condensate assembled via liquid–liquid phase separation (LLPS)^10,11^. According to current ideas, LLPS is driven by weak interactions between proteins and nucleic acids and, as a rule, involves intrinsically disordered proteins (IDPs) that contain intrinsically disordered regions (IDRs)^11,12^. In the vertebrate model species, *Xenopus* and *Danio,* the initial stages of the Bb formation depend, respectively, on Xvelo and Bucky ball proteins that contain IDRs and prion-like domains. These proteins, referred to as amyloid-forming proteins, form networks with amyloid-like properties that are capable of recruiting mitochondria and RNA molecules to the nascent Bb^10,13^. Currently, it is not known whether similar processes are also involved in the formation of the Bb in invertebrates^14^.

As the morphology and composition of the Bb may fundamentally differ even in related taxa, it is not surprising that various functions have been attributed to the Bb. It has been suggested that the Bb (a) delivers germinal granules and localized mRNAs to the vegetal oocyte cortex^1,15–17^, (b) plays a role in the formation of oocyte reserve materials^9^ or (c) participates in the transfer of mitochondria to the germ plasm and/or to the next generation^1,4^.

Mitochondria are implicated in many vital cellular processes; they contain their own genome, the mitochondrial DNA (mtDNA), and protein synthesis machinery. The most important function of mitochondria is the generation of metabolic energy, i.e., the synthesis of ATP. As the latter process leads also to the accumulation of reactive oxygen species (ROS), mtDNA is particularly vulnerable to damages (mutations) that result in severe deleterious effects including a loss of metabolic function/s and a decrease of the potential of the inner mitochondrial membrane. In somatic cells, the negative effects of ROS are counterbalanced by two processes: mitochondrial fusion and mitochondrial fission (division), jointly termed mitochondrial homeostasis or mitochondrial dynamics. Hindering mitochondrial fission results in the formation of extensive (hyperfused) mitochondrial networks, whereas hampering mitochondrial fusion maintains mitochondria in the form of individual bean-shaped organelles. Therefore, the actual morphology of mitochondria in a given cell depends on a balance between these opposed processes. Both fusion and fission are implicated in the maintenance and inheritance of mtDNA. Damaged mitochondria with low membrane potential might be rescued by fusion with “healthy” mitochondria/mitochondrial network or alternatively eliminated, via autophagy (see below), after fission, i.e., after separation from the mitochondrial network^18–21^. It is well established that fusion and fission are mediated by multi-domain GTPases belonging to the superfamily of dynamins^21,22^. Key components of the fission machinery include elements of ER, actin filaments (microfilaments, MF) that collaborate to generate initial constriction of the mitochondrion, and a highly conserved dynamin-related protein 1 (Drp1) that drives further steps of mitochondrial division^23–26^.

Interestingly, recent studies showed that the mitochondria constituting the Bb are interconnected forming extensive mitochondrial network that display distinctly higher inner membrane potential than mitochondria in the remaining part of the oocyte cytoplasm (ooplasm)^27–29^. The same studies also showed that the ooplasm surrounding the Bb often contains degenerating mitochondria^14,27,30^. Interpretation of these findings have led to the suggestion that the Bb may participate not only in the transfer of mitochondria to the next generation but also in the selective elimination of defective (containing mutated mtDNA) mitochondrial units^27,30–34^. In somatic cells, the elimination of defective mitochondria depends on autophagy (mitophagy), a complicated multistep process comprising four stages: (1) initial formation of a short, flat cisterna, referred to as autophagosome assembly site or a phagophore, (2) elongation of this cisterna and engulfment of autophagic cargo, e.g., mitochondrion, (3) closure of phagophore edges and formation of a vesicle, the autophagosome, and (4) fusion of the autophagosome with the lysosome^35^.

To gain further insight into the function of the Bb we performed a series of analyses including computer-aided 3-dimensional (3D) reconstructions of serial semithin and ultrathin sections, assessment of the potential of the inner mitochondrial membrane, detection of DNA (mtDNA) synthesis (using a thymidine analog, BrdU) as well as immunolocalization. A common bush-cricket, *Meconema meridionale,* served as a model in these studies. We selected this species because of the following reasons: (1) the Bb of *Meconema*, as these of other bush crickets^3,14^, is relatively large and easily recognizable, even at the level of light microscopy; (2) oocytes of bush crickets are morphologically simple and devoid of any specialized ooplasm regions, such as the germ/pole plasm^3,14^. The latter trait facilitates the analysis of the Bb morphogenesis and (especially) the fate of the Bb constituents after the dispersion of this organelle assemblage. It is of note to add that bush crickets, together with crickets, grass-hoppers and ground-hoppers, comprise one of the basally branching insect taxon^36^. We believe that such taxonomic nesting of the studied species, may also add some new arguments to the discussion of the evolutionary ancestral (primary) function of the Bb.

We showed that in *Meconema* oocytes the Bb forms during the meiotic prophase, in the stage referred to as the bouquet stage, and comprises (as in other hemimetabolous insects), an intricate mitochondrial network. We showed also that during mid to late previtellogenesis, this network expands filling almost entire ooplasm, then gradually partitions into small bean-shaped mitochondria. Additionally, our analyses suggest that at least some of the individual mitochondria associate with phagophores and are eliminated via mitophagy. These findings substantiate the suggestion that two important processes, i.e., multiplication of oocyte mitochondria and the selective elimination of defective (containing mutated mtDNA) mitochondrial units take place within the Bb. In this way, the Bb contributes to the inheritance of mtDNA.

## Results and discussion

### Morphology of the ovary and ovariole

**Figure 1:**
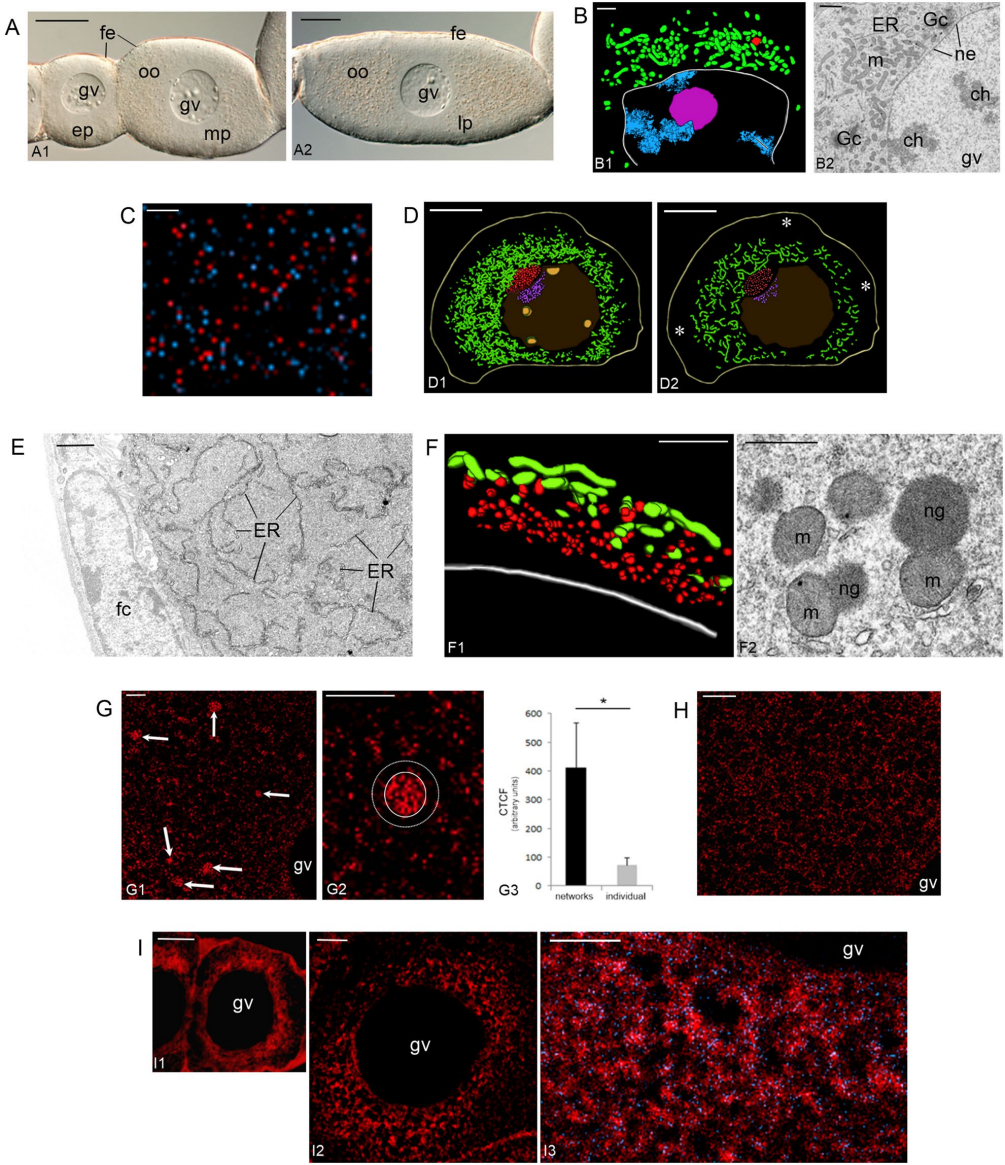
*Meconema* Balbiani body (Bb) forms in meiotic oocytes and starts to expand during early previtellogenesis. (**A**) An early- (ep), mid- (mp) (A1) and late-previtellogenic (lp) (A2) ovarian follicles of *Meconema*. Germinal vesicle (gv), follicular epithelium (fe), ooplasm (oo). Whole-mount preparations, DIC. Scale bars: 30 µm. (**B**) The Bb in bouquet stage oocyte; computer-aided 3D reconstruction (B1, based on 4 ultrathin sections) and an electron microscopy image (B2). The mitochondrial network (green, m), Golgi complexes (Gc), endoplasmic reticulum (ER), nuage aggregation (red), bouquet stage chromosomes (blue, ch), and nucleolus (magenta). The nuclear envelope (ne) of a germinal vesicle (gv) is outlined in white. Scale bars: 1 µm. (**C**) Incubation with BrdU shows intense replication of mtDNA during previtellogenesis. Paraplast section labeled with anti-BrdU antibody (red dots) and counterstained with DAPI (mtDNA, blue dots). Scale bar: 1 µm. (**D**) The Bb in an early-previtellogenic oocyte. Computer-aided 3D reconstructions based on 10 (D1) and 2 (D2) serial semi-thin sections. The Bb is surrounded by a transparent layer of peripheral ooplasm (asterisks in D2). Mitochondrial network (green), nuage aggregations (red), germinal vesicle (brown), prenuage granules (violet), and nucleoli (yellow). The oocyte plasma membrane (oolemma) is outlined in beige. Scale bars: 10 µm. (**E**) Early-previtellogenic oocyte; an electron microscopy image of the peripheral layer of ooplasm. Note the elongated cisternae of the ER and free ribosomes. Follicular cell (fc). Scale bar: 1 µm. (**F**) Early-previtellogenic oocyte; small aggregations of nuage (red, ng) associate with the Bb mitochondria (green, m). Computer-aided 3D reconstruction (F1, based on 5 ultrathin sections) and an electron microscopy image (F2). The nuclear envelope is outlined in white. See also Supplementary Movie S1. Scale bars: 1 µm. (**G**) An ooplasm of late-previtellogenic oocytes contains mitochondrial micro-networks (arrows) and individual mitochondria. Germinal vesicle (gv). Paraplast sections of ovarian tissue incubated with MitoTracker (G1, G2). An individual micro-network (encircled with a solid line in G2) is surrounded by a less fluorescent “halo” (between solid and dotted circles in G2) in which only individual less active mitochondria were present. The bar graph of quantitative image analysis of corrected total cell fluorescence (CTCF) (G3) shows that the activity of individual mitochondria (located in the vicinity of micro-networks) is lower than that of the mitochondrial units constituting micro-networks. Scale bars: 10 µm. (**H**) During final phase of late previtellogenesis, the ooplasm contains individual mitochondria only. Germinal vesicle (gv). Paraplast section of ovarian tissue incubated with MitoTracker. Scale bar: 10 µm. (**I**) Amyloid-forming proteins participate in the formation of the Bb. Paraplast sections of meiotic (I1) and an early-previtellogenic oocyte (I2, I3) stained with PROTEOSTAT Protein Aggregation Assay and counterstained with DAPI. The PROTEOSTAT-positive regions (stained red) encompass blue dots representing mtDNA (I3). Germinal vesicle (gv). Scale bars: 5 µm.

Ovaries of *Meconema meridionale* are, as in all other orthopterans, panoistic (i.e., devoid of supporting nurse cells) and composed of several, synchronously developing functional units, termed the ovarioles^3^. Fully grown ovarioles of *Meconema* comprise anteriorly located terminal filament followed by a small germarium and elongated vitellarium that consists of linearly arranged ovarian follicles. The follicles (and the oocytes contained within) are traditionally classified into 3 basic categories: previtellogenic, vitellogenic, and choriogenic (Fig. 1A).

### Morphogenesis of the Bb and differential activity of its mitochondria

Our analyses showed that in *Meconema* the Bb started to form as early as in meiotic oocytes located in the basal portion of the germarium. In a stage referred to as the bouquet stage, the Bb was easily recognizable. It was a loose organelle assemblage comprised of variously shaped, often bifurcated mitochondria, nuage aggregations, short ER cisternae, and GCs (Fig. 1B). Computer-aided 3D reconstructions of electron micrographs showed that the Bb was invariably located next to the segment of the nuclear envelope where the telomeres of the bouquet chromosomes were attached, and that the Bb mitochondria were interconnected forming a loosely arranged network (Fig. 1B1). Such organization of early meiotic Bb is characteristic of other basally branched insects^3,14,27^.

Incubation with a thymidine analog BrdU demonstrated an intense synthesis (replication) of mtDNA in previtellogenic oocytes (Fig. 1C and Supplementary Fig. S1). In this context, we counted mitochondria on TEM micrographs taken from oocytes at subsequent developmental stages (representative micrographs are shown in Supplementary Fig. S2). This quantitative comparison showed that the number of mitochondria in mid-previtellogenic oocytes was 2.4 times higher than the number of these organelles in early-previtellogenic oocytes. Similarly, the number of mitochondria contained in the ooplasm of late-previtellogenic oocytes was 2.7 times higher than in mid-previtellogenic ones. Taken together our observations showed that during previtellogenesis of *Meconema*, the oocyte mitochondria and/or mitochondrial units integrated in the Bb intensely multiplicate.

Performed computer aided 3D reconstructions of whole oocytes based on serial semi-thin sections showed that during early previtellogenesis the Bb is much larger than described earlier using incidental (not serial) sections only (see^3^ for further details). It filled almost entire ooplasm (Fig. 1D) and comprised polymorphic mitochondria, ER cisternae, GCs, lysosome-like organelles and small aggregations of nuage material (Supplementary Fig. S3). The latter are located asymmetrically (on one side of the germinal vesicle), often in a shallow depression of the nuclear envelope. This “mass” of mitochondria and associated organelles was surrounded by a conspicuous layer of ooplasm that contains ER cisternae and ribosomes only (Fig. 1D,E). Performed 3D reconstructions showed additionally that in the oocytes of *Meconema*, as it is the case in the oocytes of other hemimetabolous insects^3,14,27^, the Bb mitochondria are interconnected constituting an intricate network (Fig. 1D) and that mitochondrial units located in proximity to the nuclear envelope were often associated with nuage aggregations (Fig. 1F and Supplementary Movie S1).

Analysis of ultrathin sections at higher magnifications showed that the nucleoplasm “underlying” nuage aggregations contained distinct accumulation of spherical dense granules (Fig. 1D and Supplementary Fig. S3). Similar granules were absent in the remaining regions of the nucleoplasm (Fig. 1D). These observations suggested that the granules comprised the macromolecules participating (after translocation via nuclear pore complexes) in the formation of nuage aggregations. In this context, we termed these granules the “prenuage granules”. Interestingly, the morphologically similar granules were found to participate in the formation of nuage in the oocytes of a model organism, *Xenopus laevis*^37^.

Incubation with MitoTracker indicated that in late-previtellogenic oocytes, the mitochondrial networks became partitioned into smaller units referred to as the micro-networks (Fig. 1G1,G2). Each micro-network was surrounded by a less fluorescent “halo” in which only individual mitochondria were present (Fig. 1G2). The quantitative image analyses showed that the membrane potential (activity) of the individual mitochondria is apparently lower than that of mitochondrial units constituting micro-networks (P < 0.05) (Fig. 1G3). This result, in turn, suggests that mitochondria separated from the network are destined for degradation. As the result of the progressive disassembly of mitochondrial micro-networks and divisions of individual mitochondria (see below), during final phase of late previtellogenesis, the ooplasm was uniformly filled with single mitochondria (Fig. 1H).

### Amyloid-forming proteins participate in the formation of the Bb

To test whether amyloid-forming proteins are implicated in the formation of the Bb in *Meconema* (as they are in vertebrate model species) we used the PROTEOSTAT Protein Aggregation Assay that was previously employed to stain amyloid-like assemblies^38,39^. Analysis of stained sections revealed that amyloid-forming proteins were enriched in the vicinity of the germinal vesicle in meiotic (Fig. 1I1) and early-previtellogenic oocytes (Fig. 1I2), i.e., in the region of ooplasm where the early Bb forms. High magnification images showed that PROTEOSTAT-positive regions encompassed DAPI-positive foci representing mtDNA (Fig. 1I3). These results suggested that the initial stages of the Bb formation in *Meconema* may depend on amyloid-forming proteins (IDPs) and that these proteins could be responsible for the recruitment of mitochondria to the Bb. Both suggestions are consistent with current ideas on the Bb formation in the model vertebrates^10,11,13^. To support results obtained with PROTEOSTAT, we incubated dissected ovaries in Grace’s Insect Medium containing the aliphatic solvent 1,6-hexanediol, the reagent known to disperse (dissolve) biomolecular condensates formed by IDPs^40,41^. This treatment led to the relocation and dispersion of the Bb constituents throughout the ooplasm. Described effect was especially evident in early-previtellogenic oocytes (compare Supplementary Figs. S3 and S4) giving an additional argument for the involvement of IDPs in the early stages of *Meconema* Bb formation.

### Divisions of mitochondria: multiplication *vs* mitophagy

**Figure 2:**
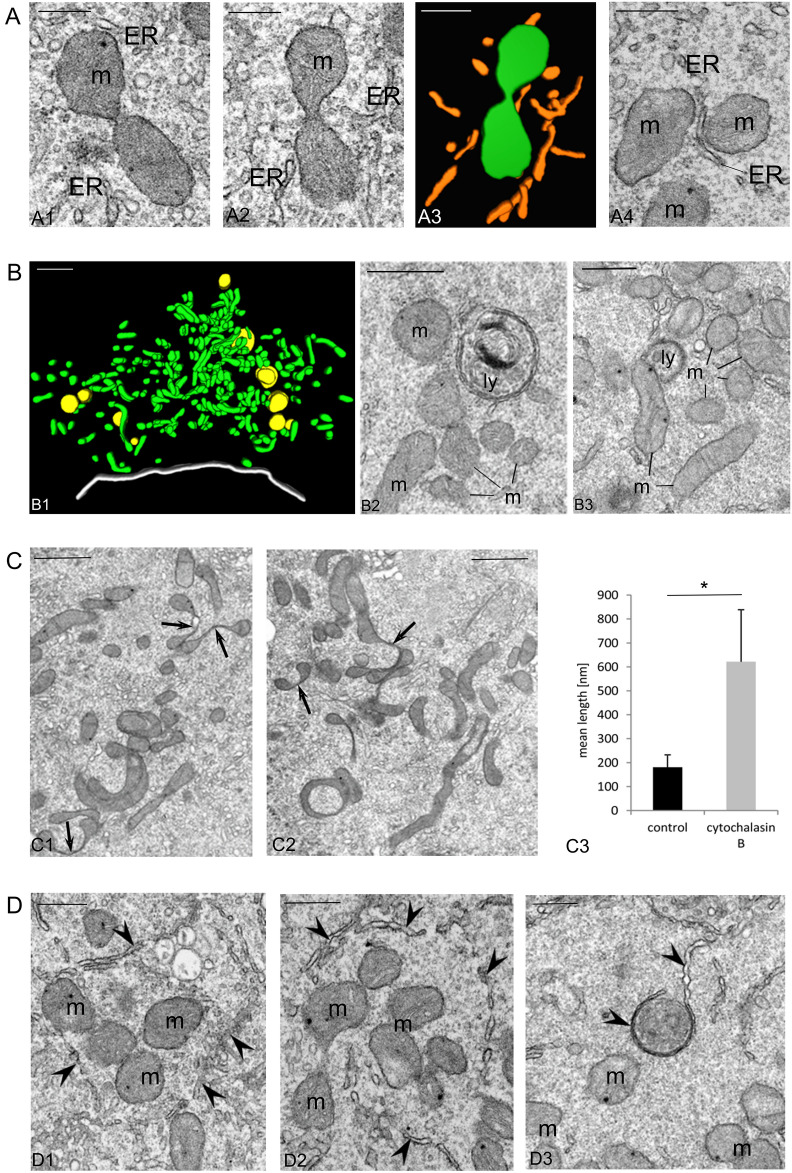
Fissions of the Bb mitochondrial network ultimately lead to the formation of individual bean-shaped mitochondria; this process depends on microfilaments, and ER elements. (**A**) ER is involved in mitochondrial divisions. Mid-previtellogenic oocyte; electron microscopy images (A1-A2, A4) and computer-aided 3D reconstruction (A3, based on 2 ultrathin sections). Note mitochondria (green, m) associated with elements of the ER (orange, ER). Scale bars: 0.5 µm. (**B**) Early-previtellogenic oocyte; the Bb mitochondria (green, m) located away from nuage aggregations associate with lysosome-like organelles (yellow, ly). Computer-aided 3D reconstruction (B1, based on same series of ultrathin sections used for reconstruction shown in 1F1) and electron microscopy images (B2, B3). The nuclear envelope is outlined in white. See also Supplementary Movie S2. Scale bars: 1 µm in B1, 0.5 µm in B2 and B3. (**C**) Mitochondrial divisions depend on microfilaments. After cytochalasin B treatment, mitochondria become elongated and atypically constricted (arrows). Mid-previtellogenic oocyte; electron microscope images (C1, C2). The bar graph of quantitative analysis (C3) indicates that the mean length of mitochondrial constrictions in cytochalasin B treated oocytes is significantly higher than the mean length of constrictions in control material (compare **A** and **C**). Scale bars: 1 µm. (**D**) Mid-previtellogenic oocyte; mitochondria (m) are often surrounded by phagophore-like structures (partly open or circular cisternae marked by arrows). Electron microscopy images (D1-D3). Scale bars: 0.5 µm.

As we showed in the section “Morphogenesis of the Bb and differential activity of its mitochondria”, during oogenesis of *Meconema*, as in all animal species^3,14^, the number of mitochondria and/or mitochondrial units integrated in mitochondrial network grows substantially. In this context, we screened TEM images searching for the dumbbell-shaped, hourglass-shaped, bifurcated, and merged mitochondria, plausibly exemplifying mitochondria in consecutive steps of constriction and scission. We found that such images were relatively frequent (Fig. 2A). Further analyses of images, and 3D reconstructions revealed additionally that constricted mitochondria were associated with ER cisternae (Fig. 2A3) or, alternatively, with lysosome-like organelles (Fig. 2B and Supplementary Movie S2). This observation support a recent model assuming that there are at least two types of mitochondrial divisions. The first involves Drp1 and lysosomes and leads to the separation of inactive mitochondrial units destined for mitophagy; the second involves Drp1, ER elements, and MFs, and enables the multiplication of “healthy” mitochondria^25^.

In this context, we asked whether MFs participate in the divisions of the Bb mitochondria. To answer this question, we incubated the ovaries of *Meconema* in Grace’s Insect Medium supplemented with cytochalasin B. This mycotoxin inhibits actin polymerization as it binds to MFs barbed (plus) ends. Such treatment had no visible effect on whole oocytes and their morphology. However, it revealed that the mitochondria of treated oocytes were strongly elongated, clustered into irregular aggregates, and often atypically constricted (compare Fig. 2C1,C2 with Fig. 2A and Supplementary Fig. S3). Performed measurements demonstrated that the mitochondrial constrictions observed in cytochalasin B treated oocytes were longer (mean length 621 nm) than the constrictions in control material (mean length 181 nm) (Fig. 2C3). Such abnormal constrictions displayed striking resemblances to elongated “stalled” super-constrictions observed in mammalian cell lines in which mitochondrial divisions were experimentally hampered^42^. These data suggest that divisions of the Bb mitochondria depend on intact MFs.

Finally, we screened our images searching for ultrastructural evidence of mitophagy. We found that some mitochondria were associated with lysosome-like organelles (Fig. 2B and Supplementary Movie S2). Furthermore, some mitochondria displaying signs of degeneration were surrounded by partly open or closed circular cisternae (Fig. 2D). We interpreted these images as sections of developing/growing phagophores.

Overall, our results suggest that both types of mitochondrial divisions described in^25^, the one leading to mitochondria proliferation and the other terminating in mitophagy, take place in the cytoplasm of *Meconema* oocytes.

### Microtubules participate in the positioning of the Bb constituents

**Figure 3:**
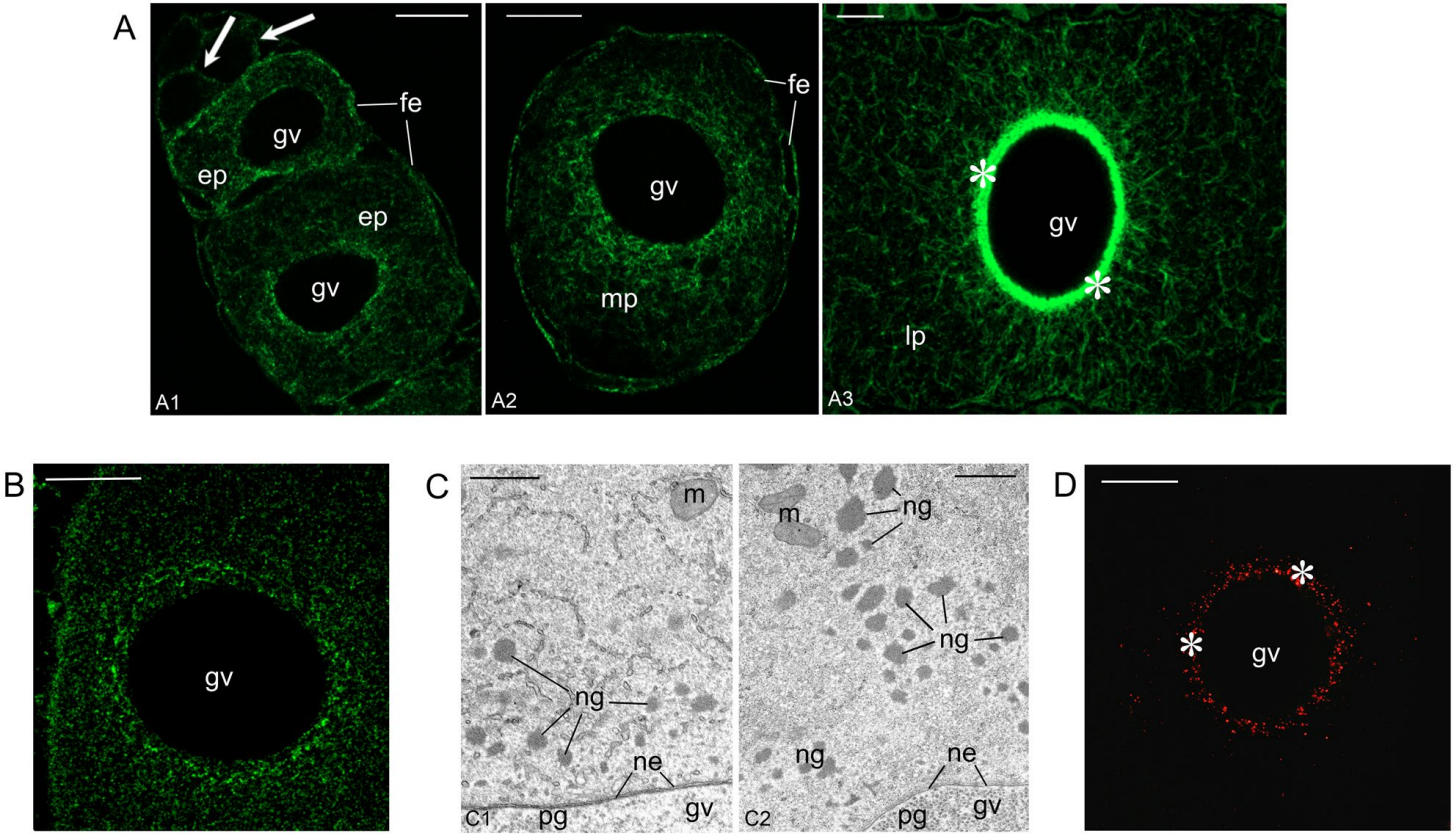
Elaborate system of microtubules participates in the positioning of nuage aggregates next to the nuclear envelope. (**A**) Distribution of microtubules alters during oogenesis. In meiotic (arrow) and early-previtellogenic oocytes (ep) (A1) microtubules are relatively rare and more or less evenly distributed. In mid-previtellogenic (mp) oocytes (A2) microtubules are more abundant in the perinuclear ooplasm. Finally, in late-previtellogenic oocytes (lp) (A3) microtubules form a recognizable layer (asterisks) around the germinal vesicle. Follicular epithelium (fe), germinal vesicle (gv). Paraplast sections stained with an antibody against α-tubulin. Scale bars: 10 µm. (**B**) Late-previtellogenic oocyte; colchicine treatment disrupts the microtubular layer around germinal vesicle (gv) and radiating microtubules. Paraplast section stained with an antibody against α-tubulin. Scale bar: 10 µm. (**C**) Late-previtellogenic oocyte; colchicine treatment leads to mislocalization of nuage aggregations. Electron microscopy images. (C1) Control oocyte; nuage aggregations are located next to the nuclear envelope. (C2) Oocyte incubated with colchicine; nuage aggregations are located away from the nuclear envelope. Germinal vesicle (gv), mitochondria (m), nuclear envelope (ne), nuage aggregations (ng), prenuage granules (pg). Scale bars: 1 µm. (**D**) Late-previtellogenic oocyte. Polyglutamylated tubulin is more frequent in the perinuclear layer (asterisks) than in radiating microtubules. Paraplast section stained with an antibody against polyglutamylated subunits of tubulin. Germinal vesicle (gv). Scale bar: 20 µm.

Asking whether microtubules participate in the formation and/or integrity of the Bb, we first stained paraplast sections with an antibody against α-tubulin. We revealed that the distribution of microtubules altered as oogenesis progressed. Initially (in meiotic and early-previtellogenic oocytes) microtubules were relatively rare and evenly distributed (Fig. 3A1). During mid previtellogenesis they were more abundant in the perinuclear ooplasm (Fig. 3A2). Finally, in late-previtellogenic oocytes, microtubules gathered around the germinal vesicle forming there an easily recognizable layer (Fig. 3A3). Additional, individual microtubules radiated from this layer towards the oocyte periphery (Fig. 3A3).

Next, we incubated *Meconema* ovaries in Grace’s Insect Medium supplemented with colchicine. This experiment showed that 24-h exposure to colchicine disrupted all oocyte microtubules, and staining with an antibody against α-tubulin revealed only a patchy, fluorescent “halo” around the germinal vesicle (Fig. 3B). TEM analysis showed that the colchicine treatment led to the mislocalization of mitochondrial units (constituting the mitochondrial network) and nuage aggregations (Fig. 3C); the latter did not reside next to the nuclear envelope (as it is in untreated oocytes; Fig. 3C1) but were located away from the germinal vesicle (Fig. 3C2). On the basis of these observations, we concluded that the perinuclear microtubular layer participates in the positioning of the important Bb constituents in the close vicinity of the nucleus.

The degree of glutamylation/polyglutamylation of microtubules, or more precisely tubulin subunits, may (indirectly) specify the position of cytoplasmic organelles^43^. Therefore, we asked whether microtubules present in *Meconema* oocytes are differentially glutamylated, and stained sections with an antibody recognizing polyglutamylated subunits of tubulin. This staining revealed that subunits of polyglutamylated tubulin were more abundant in the perinuclear layer (where microtubules were more frequent) than in peripherally located “radiating” microtubules (Fig. 3D). This result provided additional support to the suggestion that the microtubules participate in the positioning of the Bb constituents.

Finally, it should be mentioned that clusters of mitochondria, termed mitoballs, have recently been described in premeiotic spermatocytes of several insect species, including *Drosophila melanogaster*^44^. Like the Bbs, the mitoballs are transient aggregations and may comprise, in addition to mitochondria, other organelles, such as ER elements and GCs. Moreover, it has been shown that in *D. melanogaster* the formation of the mitoball in spermatocytes^44^ and the Bb^45^ requires Milton, an adaptor protein linking mitochondria to microtubules via kinesin heavy chain. Despite these similarities, the mitoball and the Bb differ functionally: the mitoball is not necessary either for the replication of mtDNA or for the elimination of dysfunctional mitochondria^44^. Apparent functional differences between the mitoball and the Bb raise an intriguing question: how is the functioning of morphologically alike mitochondrial clusters regulated in male *versus* female gonads. Although we are far from answering this question, we would like to stress that the Bb and the mitoball are easily distinguishable by one morphological feature: the nuage is a constant element of the Bb, whereas it is absent in the mitoball. It is tempting to speculate, in this context, that the nuage contains macromolecules indispensable for the functioning of the female mitochondrial cluster, the Bb.

## Conclusions

1. Our results provide further evidences for the involvement of the Bb in the multiplication and selection of mitochondria in female germline cells.
2. The formation of *Meconema* Bb involves amyloid-forming proteins, indicating the homology of vertebrate and invertebrate Bb and, therefore, the common evolutionary origin of this organelle assemblage.
3. During subsequent stages of oogenesis, the integrity of the Bb as well as positioning of its elements (next to germinal vesicle) depend on microtubular cytoskeleton.
4. The interpretation of our results in a phylogenetic context supports the previously voiced idea^3,14,30^ that the transmission of mitochondria to the offspring represents an ancestral function of the Bb. Other functions attributed to the Bb seem to be evolutionary “younger”; we postulate that they evolved independently in derived animal groups (frogs, holometabolous insects, and certain chelicerates) secondarily.

## Materials and methods

### Animals

Dozens of individuals of the bush cricket, *Meconema meridionale* (Tettigoniidae, Meconematinae) were collected in the neighborhood of Krakow, Poland (50°03ʹ41″N 19°56ʹ14″E) and settled in our experimental garden. This species has become relatively common, and is neither protected nor endangered, therefore no specific permits are required. The colony, maintained for three years, reproduced successfully after each winter season. Last instar female larvae and young females were obtained from this colony during June and July. They were anesthetized and killed by decapitation. For each experiment, 5–10 specimens were used; each analysis was repeated at least three times.

### Light and electron microscopy

The ovaries were dissected under a Nikon SMZ 1500 stereomicroscope (Nikon, Japan). Gonads were fixed in a mixture of 2% formaldehyde (Sigma–Aldrich) and 2.5% glutaraldehyde (Sigma–Aldrich) in 0.1 M phosphate buffer (pH 7.3). After several weeks, samples were rinsed in phosphate buffer and post-fixed in a mixture of 1% osmium tetroxide (Sigma-Aldrich) and 0.8% potassium ferrocyanide (Chempur, Poland) for 60 min at 4 °C. After dehydration in the graded series of ethanol and acetone, the material was infiltrated in a mixture of acetone and Epoxy Embedding Medium (Sigma-Aldrich), placed in a vacuum drier (Thermo Fisher Scientific) for three hours and embedded in Epoxy Embedding Medium. Semithin sections (0.7–1 μm thick) were stained with 1% methylene blue and examined under a Leica DMR light microscope (LM) (Heidelberg, Germany). Ultrathin sections (80 nm thick) were contrasted with uranyl acetate and lead citrate according to standard protocols and analyzed with a transmission electron microscope (TEM) Jeol JEM 2100 (Tokyo, Japan) at 80 kV. To present larger fragments of oocytes some images were composed by combining several microphotographs using the Image Composite Editor software. For measurements we used either specific software of the Jeol JEM 2100 microscope or the Image J (NIH) program.

### 3D organization of the Bb and its constituents

To reconstruct the three-dimensional (3D) organization of the Bb serial semithin or ultrathin sections were used. Microphotographs were aligned to form virtual stacks and required structures (mitochondria, nuage accumulations, degenerating mitochondria, lysosomes) were contoured using the TrakEM2 plugin of the ImageJ/Fiji Software (NIH). 3D reconstructions were created using the 3D viewer and Z-projections plugins of the same program.

### Immunohistochemistry

Dissected ovaries were fixed in 4% formaldehyde (PFA) in PBS at room temperature (RT) for 1 h. After dehydration through a series of ethanol and HistoChoice Clearing Agent (Sigma-Aldrich) samples were embedded in the paraplast. 3 to 5 μm sections were cut, mounted on microscopic slides, deparaffinized, rehydrated, and rinsed in water. The sections were then permeabilized with PBS containing 0.05% Tween 20 (Sigma-Aldrich) and 0.1% Triton X-100. Non-specific binding sites were blocked in blocking buffer (Blocker Casein in PBS (Thermo Scientific) and 0.05% Tween 20) for 3 h at RT. The sections were then incubated overnight at 4 °C with primary antibodies (for the list of antibodies see Table 1). On the next day, secondary goat anti-mouse or goat anti-rabbit antibodies (Table 1) were applied. For the negative control, the respective primary antibody was omitted and no fluorescence was observed. The stained sections were mounted in ProLong Gold antifade reagent with DAPI (Invitrogen) and imaged under a DMR Leica epifluorescence microscope (FM) and a Zeiss LSM 900 confocal microscope. The images were analyzed using ImageJ Software.

**Table 1:** The list of primary and secondary antibodies.

Antibody	Host species	Vendor	Cat. number	Dilution	RRID
Primary antibodies					
Anti-α-Tubulin	Mouse	Sigma-Aldrich	T5168	1:2000	AB_477579
Anti-PolyG	Mouse	AdipoGen	AG-20B-0020	1:200	AB_2490211
Anti-BrdU FITC conjugated	Mouse	Millipore	MAB3262F	1:50	AB_94398
Secondary antibodies					
Alexa Fluor 488 anti-mouse	Goat	Thermo Fisher Scientific	A11001	1:200	AB_2534069
Cy3 anti-mouse	Goat	Thermo Fisher Scientific	A10521	1:200	AB_10373848

*PolyG* polyglutamylation modification of tubulin, *RRID* research resource identifier.

### Detection of protein aggregates

Dissected ovaries were fixed in 4% PFA in PBS (1 h, RT), embedded in paraplast, and sectioned as described above. The protein aggregates were detected using the PROTEOSTAT Protein Aggregation Assay. The sections were deparaffinized, rehydrated, permeabilized (see above), and stained for 30 min, at a final concentration of 1:2000. Staining was performed adapting manufacturer’s instructions (PROTEOSTAT Protein Aggregation Assay, ENZ-51023- KP050) to tissue sections. The stained sections were mounted in ProLong Gold antifade reagent and imaged under a DMR Leica epifluorescence microscope and a Zeiss LSM 900 confocal microscope. The images were analyzed using ImageJ Software.

### Hexanediol treatment

Dissected ovaries were incubated in Grace’s Insect Medium (Sigma-Aldrich) containing 5% 1,6-hexanediol (Sigma-Aldrich, 240117) for 10 min at RT. After incubation, the ovaries were fixed in a mixture of 2% formaldehyde (Sigma–Aldrich) and 2.5% glutaraldehyde (Sigma–Aldrich), embedded in Epoxy Embedding Medium and examined as described in the section *Light and electron microscopy*.

### Colchicine and cytochalasin B treatment

Dissected ovaries were incubated in Grace’s Insect Medium containing colchicine (Sigma-Aldrich, C975) or cytochalasin B (Sigma-Aldrich, C2743) for 24 h at RT. The final stock solution (10 mg/ml) of colchicine was diluted 1:20 with Grace’s Insect Medium. Similarly, the stock solution of cytochalasin B was diluted 1:500 with Grace’s Insect Medium. After incubation, the ovaries were fixed and embedded in an Epoxy Embedding Medium or in a paraplast. Sections were stained and examined as described in the section *Light and electron microscopy*.

### Incubation with BrdU

Dissected ovaries were incubated in Grace’s Insect Medium containing 2,5-bromo-2ʹdeoxyuridine (Sigma-Aldrich, B5002) for 24 h at RT. The stock solution was prepared by dissolving 100 mg BrdU in 5 ml distilled water. To get the final concentration of BrdU, the stock solution was diluted 1:2 with Grace’s Insect Medium. After the incubation ovaries were fixed in 4% PFA in PBS (1 h, RT), embedded in paraplast, and sectioned as described above. Sections were deparaffinized, permeabilized, and blocked in a blocking buffer. Then the sections were incubated overnight at 4 °C with mouse anti-BrdU FITC conjugated antibody (Table 1)**.** The following day, the sections were mounted in ProLong Gold antifade reagent with DAPI and imaged using a Zeiss LSM 900 confocal microscope. The images were analyzed with ImageJ Software.

### Mitochondria number assessment

Mitochondria were counted on incidental ultrathin sections through the central region of ooplasm of early-, mid- and late-previtellogenic oocytes. Counting areas equaled 100 μm^2^. For each stage 15 countings were performed. Calculated mean values were multiplied by appropriate factors related to the volume of the oocyte in a given stage.

### Analysis of mitochondrial activity

The ovaries were dissected in Grace’s Insect Medium and incubated with MitoTracker Deep Red FM (Invitrogen, M22426) for 30 min at 37 °C in the dark. MitoTracker stock solution was prepared by dissolving 50 μg MitoTracker in 50 ml DMSO. To get the final concentration, the stock solution was diluted 1:200 with Grace’s Insect Medium. After incubation, the ovaries were washed twice, fixed in 4% PFA in PBS for 1 h (RT), and embedded in the paraplast as described above. Tissue sections were deparaffinized, rehydrated, mounted in ProLong Gold antifade reagent with DAPI, and imaged under a Zeiss LSM 900 confocal microscope. The images were analyzed using ImageJ Software. Mitochondrial micro-networks were outlined manually and their area, integrated density and mean grey value were measured. The same procedure was applied to individual mitochondria located in the less-fluorescent “halo” surrounding each micro-network. The corrected total cell fluorescence (CTCF) has been calculated using the following equation CTCF = area of selected fragment of the oocyte × mean fluorescence of background readings.

### Statistical analyses

The raw data were processed using Statistica 13 software (StatSoft Inc). Standard deviation (SD) and standard error of the mean (SEM) were calculated for each value. Shapiro–Wilk test was used to check normality of data distribution and Levene’s test to assess homogeneity of variance. All comparisons of means were performed using the Mann–Whitney U test. Data were considered statistically significant at *P < 0.05.

### Supplementary Information

Supplementary Video 1.

Supplementary Video 2.

Supplementary Figures.

## Data Availability

The data presented in this study are available on request from the corresponding authors.
