# Supplementary material for: Balbiani body of basal insects is potentially involved in multiplication and selective elimination of mitochondria
Source: Sci Rep. 2024 Apr 9;14:8263. doi: 10.1038/s41598-024-58997-6 (PMC11004008; doi:10.1038/s41598-024-58997-6)
Supplement: Supplementary file 3 — Supplementary Figures. [file 41598_2024_58997_MOESM3_ESM.pdf]

Balbiani body of basal insects is potentially involved in multiplication and selective elimination of mitochondria

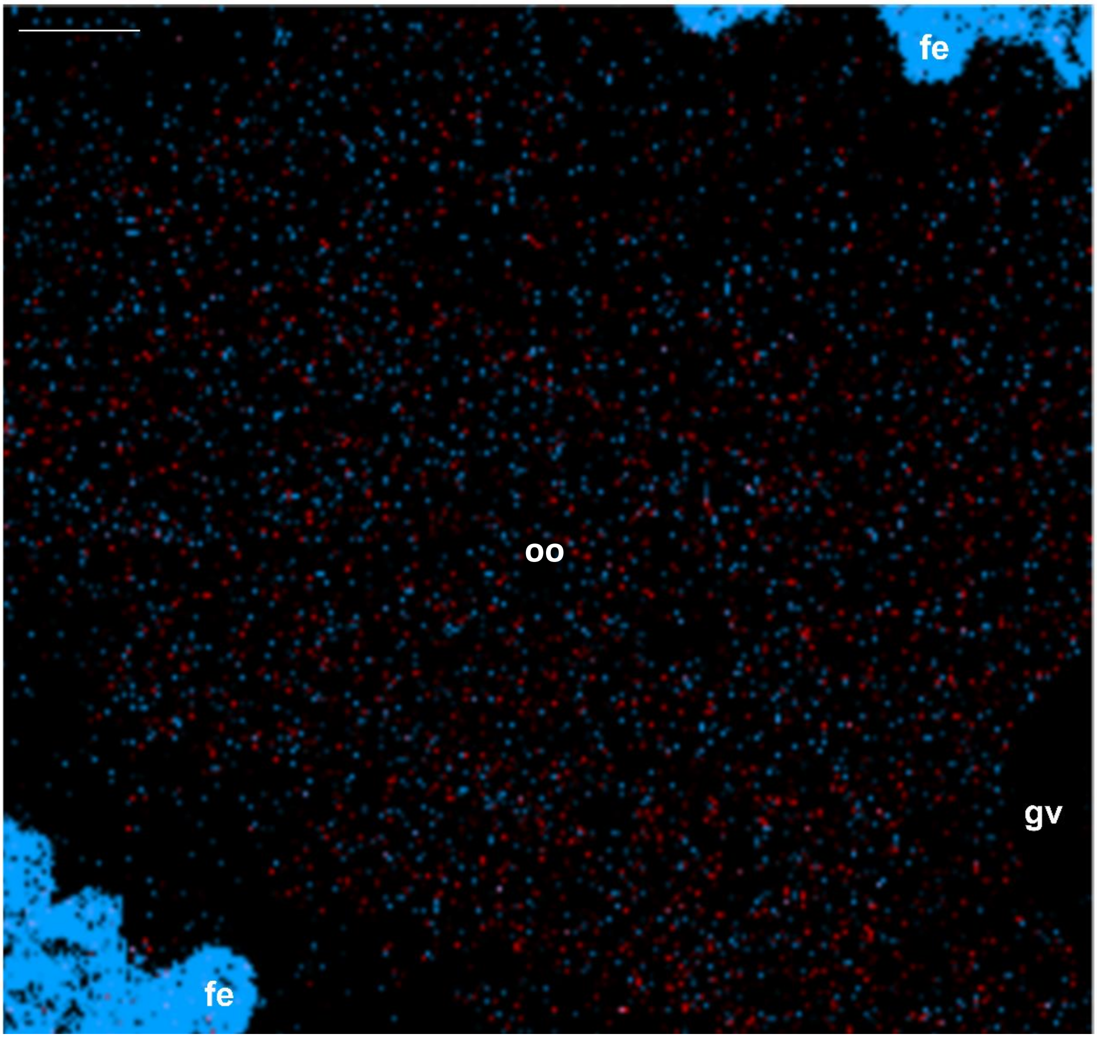

Supplementary Fig. S1. Incubation with BrdU reveals that mtDNA is abundantly replicated in the cytoplasm of mid-previtellogenic oocyte. Low magnification showing large area of the ooplasm (oo); details are shown in Fig. 1C. Note uniform distribution of replication events throughout the ooplasm. Follicular epithelium (fe), germinal vesicle (gv). Paraplast section labeled with anti-BrdU antibody (red) and counterstained with DAPI (blue). Scale bar: 20 µm.

early previtellonenesis

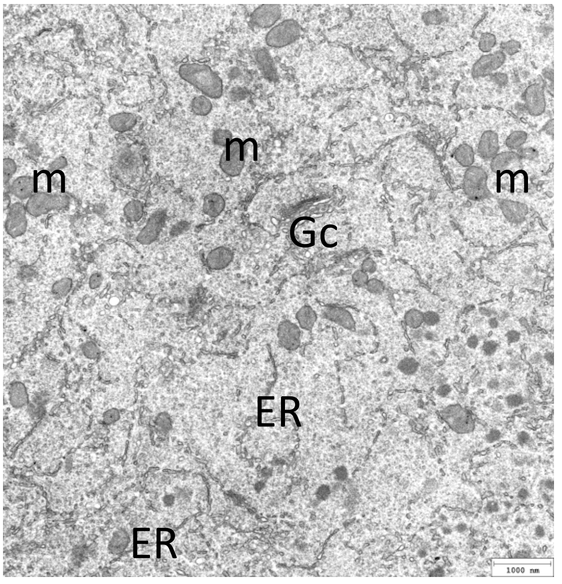

mid previtellogenesis

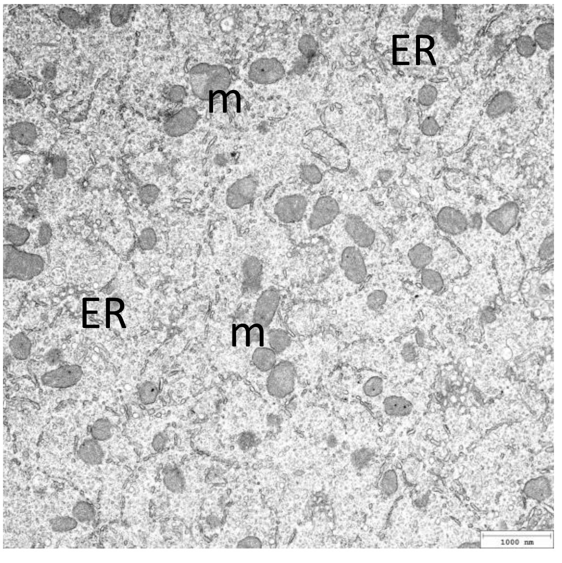

late previtellogenesis

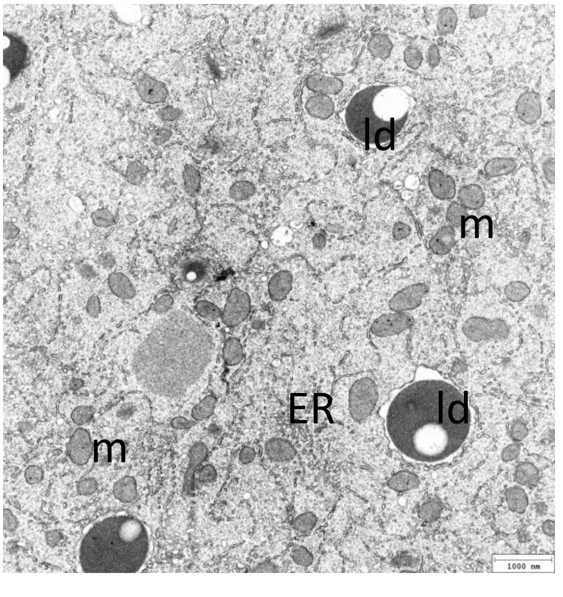

Supplementary Fig. S2. Three representative micrographs used in the mitochondrial number assessment. 100 μm^2 area of the ooplasm in early-, mid- and late-previtellogenic oocytes. ER elements (ER), Golgi complex (Gc), mitochondria (m), lipid droplets (ld) Scale bars: 1 μm.

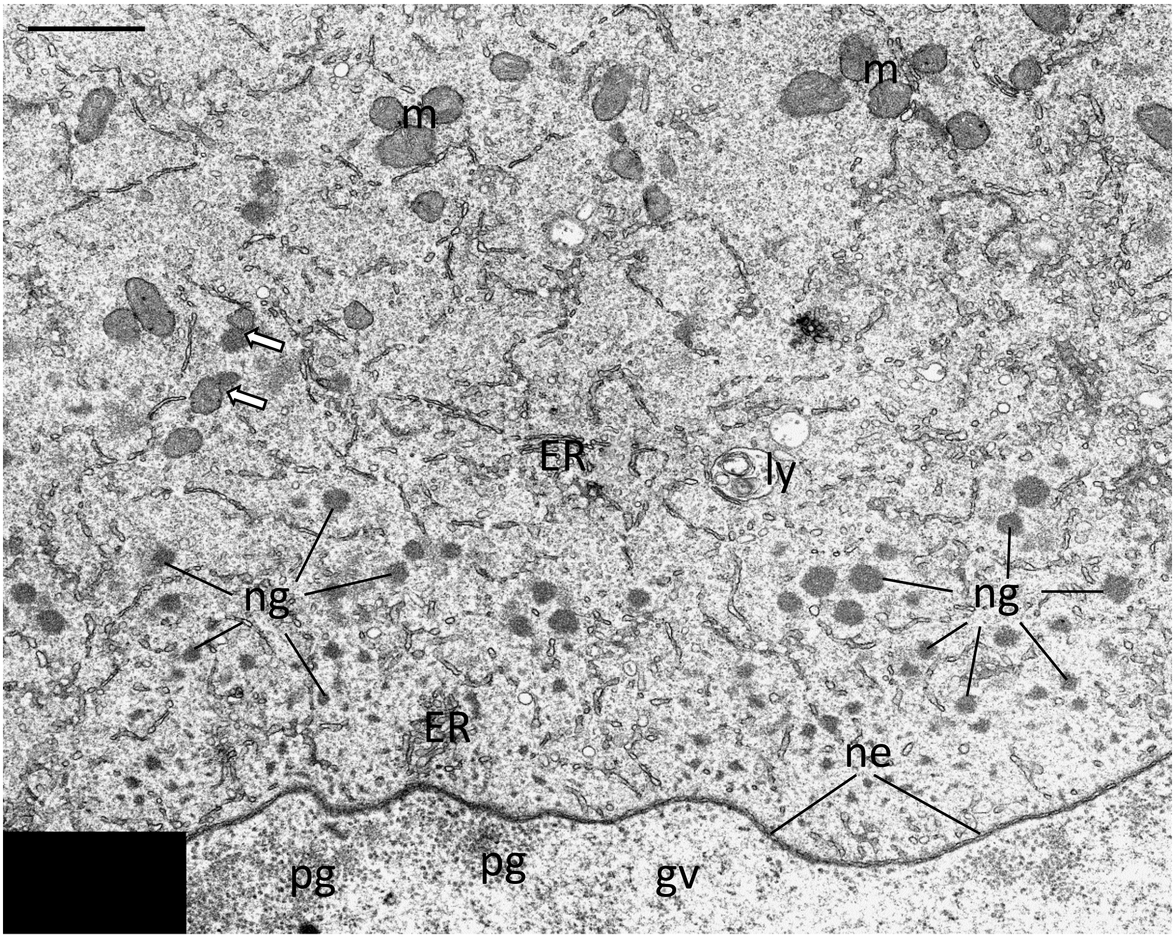

Supplementary Fig. S3. Electron microscopic image of perinuclear ooplasm and peripheral zone of the germinal vesicle (gv) of an early-previtellogenic *Meconema* oocyte. Image composed of 3 TEM micrographs. ER elements (ER), mitochondria (m), nuclear envelope (ne), nuage aggregations (ng), lysosome (ly), prenuage granules (pg). Mitochondria in a direct contact with nuage are marked with white arrows. Scale bar: 1 μm.

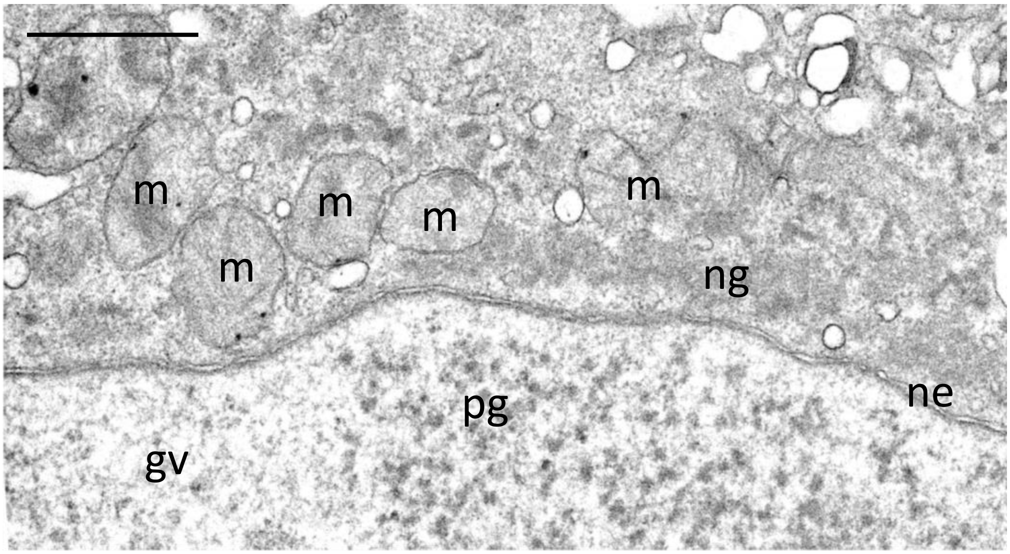

Supplementary Fig. S4. Hexanediol treatment leads to the dispersion of the Bb and mislocalization of its elements. An electron microscopic image. Note the Bb mitochondria (m) located next to the germinal vesicle (gv). Nuclear envelope (ne), nuage aggregations (ng), prenuage granules (pg). Scale bar: 1 μm.

Supplementary Movie S1. Early-previtellogenic oocyte. Computer-aided 3D reconstruction of the Bb mitochondria associated with particles of nuage. Mitochondria (green), nuage (red). The nuclear envelope is outlined in white.

Supplementary Movie S2. Early-previtellogenic oocyte. Computer-aided 3D reconstruction of the Bb mitochondria distant from nuage aggregations associated with lysosome-like organelles. Mitochondria (green), lysosome-like organelles (yellow). The nuclear envelope is outlined in white.
